# Management of undifferentiated high‐grade pleomorphic sarcoma of parotid region in elderly women

**DOI:** 10.1002/ccr3.1951

**Published:** 2019-02-07

**Authors:** Michele Sessa, Daniela Tonni, Adriano Zangrandi, Luca Oscar Redaelli de Zinis, Domenico Cuda

**Affiliations:** ^1^ Department of Otorhinolaryngology G. da Saliceto Hospital Piacenza Italy; ^2^ Department of Pediatric Otolaryngology Head Neck Surgery Children Hospital “ASST Spedali Civili” Brescia Italy; ^3^ Department of Oncology and Hematology G. da Saliceto Hospital Piacenza Italy

**Keywords:** malignant fibrous histiocytoma, management, parotid, Undifferentiated high‐grade pleomorphic sarcoma

## Abstract

Undifferentiated high‐grade pleomorphic sarcoma is a slow‐growing tumor rarely localized in the head and neck region. The treatment of UHPS should be based on large surgical resections in free margins associated with neck dissection. Postoperative radiotherapy improves local control of the disease and the prognosis quod vitam.

## 1 INTRODUCTION

Undifferentiated high‐grade pleomorphic sarcoma (UHPS), in the past known as malignant fibrous histiocytoma (MFH), represents about 5% of adult soft tissue sarcoma.^1^ UHPS most commonly occurs in the metaphysis of long bones of extremities, trunk, and retroperitoneum.^2^ Head and neck localization is very rare, from 1% to 3% of all undifferentiated pleomorphic sarcomas.^3^ In this region, the most common site is the sinonasal tract, accounting for 30%, while the parotid gland is primarily involved in 10% of cases.^4^ Lymph node metastases are present in 10%‐18% of cases, while distant metastases are reported to be as high as 42%.^4^ Surgery represents the primary modality of therapy when the lesion is resectable: en bloc resection with wide margins is the preferred strategy.^2^ Postoperative radiation therapy plays an important role in the management of this tumor in improving the local control rate_, _and the use of chemotherapy in patients with advanced stage is worthy of further investigation.^5^ We present a rare case of UHPS of the parotid region in an 84‐year‐old woman along with a review of the literature.

## 2 CASE REPORT

**Figure 1:**
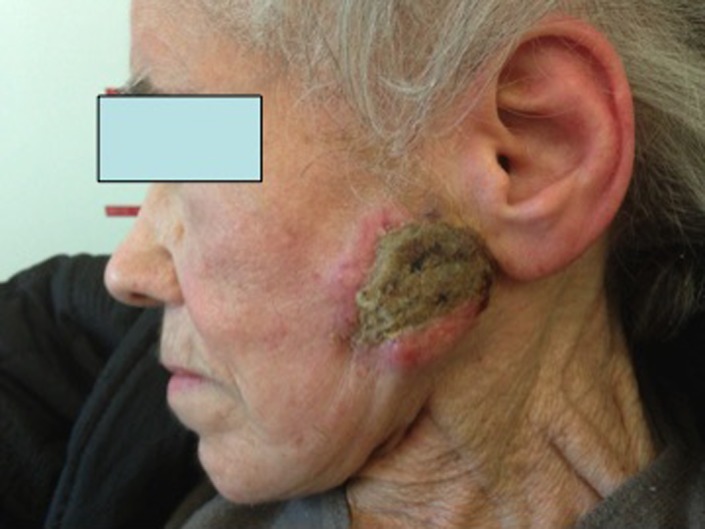
Ulcerated skin lesion of the left parotid

An 84‐year‐old woman referred to ENT Department of “Guglielmo da Saliceto” Hospital of Piacenza for a slowly progressing recurrent lesion from the skin with initial ulceration of the left parotid region detected six months before (Figure 1). The previous year, the patient presented a small nodular lesion in the same region. She was submitted to enucloresection in another institution. Histological examination showed the presence of a pleomorphic sarcoma. The deep resection margin was close to the lesion.

Physical examination revealed a solid mass with a maximum diameter of 5 cm without palpable regional lymph nodes and Grade 3 facial palsy according to the House‐Brackmann classification. Neck MRI showed a soft tissue mass of the parotid gland measuring 3.8 × 4.3 cm. The lesion infiltrated the residual part of salivary gland, the common branch of the facial nerve, the skin of the face, and the masseter muscle. Total body CT excluded macroscopic distant metastases.

A radical parotidectomy with sacrifice of the facial nerve, extended to the skin and masseter muscle, and selective neck dissection (level Ib‐II‐III) were performed. Reconstruction of the facial region was performed using a radial fasciocutaneous free flap. No primary reconstruction of facial nerve was performed considering the poor prognosis and advanced age of the patient.

**Figure 2:**
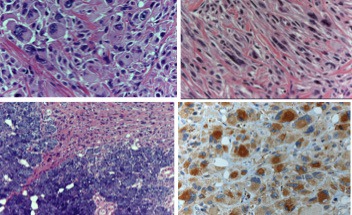
Histopatology of UHPF

The surgical specimen showed an ulcerated 5 cm large neoplasm. Soft tissues were infiltrated with a thickness of 1.5 cm. The tumor was composed of short interlacing bundles of large spindle and round cells exhibiting severe nuclear pleomorphism with scattered anaplastic cells and frequent mitoses (more than 10 mitoses/10 HPF). The tumor extended into the parotid gland, subcutaneous fat, and skeletal muscle with infiltrating borders. At immunohistochemistry, tumor cells were positive for vimentin and CD68 (both KP1 and PG‐M1 clones). Desmin, human caldesmon, smooth muscle actin, S100 protein, and cytokeratins (clone AE1/AE3) were negative (Figure 2). Surgical margins were negative. No lymph node metastases were detected.

**Figure 3:**
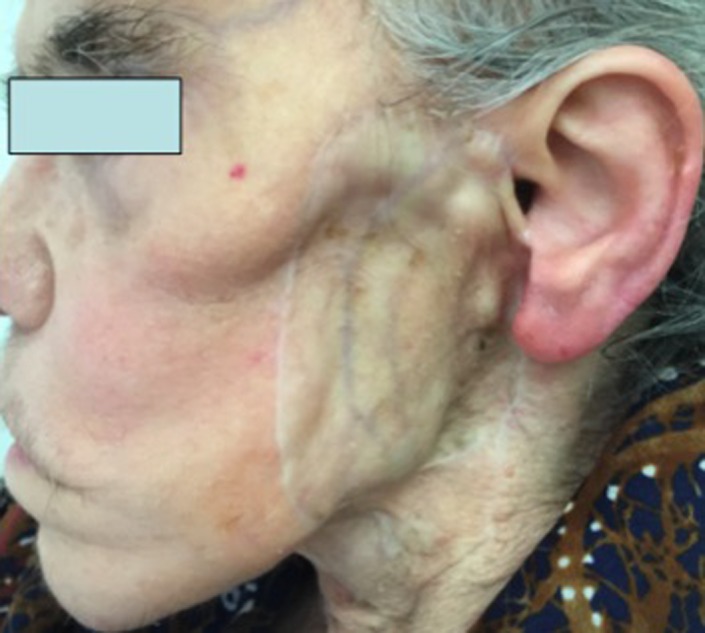
Follow‐up at 5 years

The postoperative course was uneventful. Adjuvant radiotherapy was administered on the facial region starting 4 weeks after surgery for a total dosage of 60 Gy. The patient is free of disease 5 years later (Figure 3). Informed consent was obtained from the patient to publish her case.

## 3 DISCUSSION

Undifferentiated high‐grade pleomorphic sarcoma is a soft tissue sarcoma without a definable line of differentiation. It was first described by O'Brien and Stout with the name of malignant fibrous xanthoma.^6^ The concept of UHPS has gained recognition and acceptance only in relatively recent times, having been introduced in the 2006 edition of the WHO Classification of Tumors of Soft Tissue and Bone and confirmed in the 2013 edition_._ 1, 7 Before that date, these neoplasms were classified as MFH, storiform‐pleomorphic type, according to the original description of O'Brien and Stout, and were in fact the most commonly diagnosed soft tissue sarcoma in adults. The definition implied that tumor cells exhibit some grade of “fibrohistiocytic” differentiation, as confirmed by morphology and by CD68 positivity. Many authors have reported growing evidence (from morphology, immunohistochemistry, electron microscopy, and molecular biology) that “fibrohistiocytic differentiation” does not exist and that the features originally described for these tumors may be common to many undifferentiated neoplasms (leiomyosarcoma, malignant peripheral nerve sheath tumor, liposarcoma, etc)_._ 8 With biotechnology providing more powerful tools, now it is possible to recognize subtler signs of differentiation toward specific lineages and, accordingly, to classify more properly a large number of the tumors originally termed MFH. Any signs of specific differentiation are still not detected in a small number of tumors. A small number of tumors remain in which we are not hither to able to detect any signs of specific differentiation. For these tumors, the noncommittal term UHPS is now considered appropriate and must therefore be considered a diagnosis of exclusion.^8^ UHPS are considered aggressive sarcomas with a slow pattern of growth and exhibit extensive involvement of adjacent visceral and neurovascular structures. Nodal metastases are uncommon, while distant metastases occur often. The most common localization of distant metastases is the lungs followed by bone, liver, and brain.^2^, ^4^

The etiology of UHPS is unknown but genetic background, previous radiotherapy, and traumatic burn injuries have been reported to be involved in the pathogenesis of this malignant tumor.^2^, ^3^ Head and neck localizations are very rare, their prevalence is about 1:1 000 000.^3^ UHPS usually develops in older patients with a peak incidence in the seventh decade, but have also been reported in children.^9^ A review of English language literature identified 24 published cases of primary locations of UHPS in the parotid gland (Table 1). UHPS is more frequent in adults and is very rare in early childhood^4^ as found in the literature where only 2 of the 24 cases described occurred in childhood. The analysis of the cases described in Table 1 shows a peak incidence in the sixth decade with a range from 1 year to 91 years. Males are more often affected than women by a ratio of 3 to 1. The small number of cases and the variability of treatment with highly variable follow‐up does not allow a proper analysis of data about the survival of the disease. In many cases, it presents as a steadily slow‐growing painless subcutaneous mass. An atypical rapid growth was observed in the case described by Garcia in 2008.^10^

**Table 1:** Review of literature

Author	Year	Age and sex		Treatment	Follow‐up
O'brien	1964	65 male		Exision gland	Well at 9 months
Junaid	1975	50 male		Partial parotidectomy	Local recurrence at 6 year
Jahrsdoerfer	1976	63 male		Exision gland, RT	Died with disease
Shapshay	1979	16 months male		Total parotidectomy	ND
Blitzer	1981	46 female		Exision gland	Well at 16 year
Ferrari	1982	ND		ND	ND
Benjamin	1982	28 female		Superficial parotidectomy	Well at 5 months
Wingerden	1986	41 male		Parotidectomy, RT	RT is undergoing
Auclair	1986		91 female	Superficial parotidectomy	Dead of other causes at 2 year
Auclair	1986		67 male	Superficial parotidectomy	ND
Barnes	1988		66 male	Wide excision	Dead of other causes at 5.8 year
Barnes	1988		25 male	Radical parotidectomy	well at 38 months
Schrader	1989		76 female	excison	Dead with disease at 31 months
Frankenthaler	1990		ND	ND	ND
Frankenthaler	1990		ND	ND	ND
Luna	1991		75 male	Radical parotidectomy,RT,CHT	Dead with disease at 2.6 year
Luna	1991		52 male	Radical parotidectomy,RT,CHT	Dead with disease at 3 year
Luna	1991		29 male	Total parotidectomy,RT, CHT	Local recurrence at 5 year
Wiley	1992		50 male	Parotidectomy	Well at 13 months
Odell	1996		56 male	Wide excision, RT	Dead with disease at 3 year
Venkateswaran	2000		29 male	Local resection	Well at 6.75 year
Sachse	2005		57 male	Radical parotidectomy, neck dissection, RT, CHT	Well at 10 months
Sachse	2005		54 male	Radical parotidectomy,RT	Dead with disease at 1 year
Sachse	2005		96 male	Superficial parotidectomy	Local recurrence at 3 months
Macak	2007		63 female	CHT	Died during CHT
Garcia	2008		84 female	Superficial parotidectomy,RT	Well at 1 year
Ghang	2008		6 male	CHT +RT	Well at 6 months

The management of UHPS of the head and neck is guided by stage, location, size, and patient age.MRI is essential for preoperative staging and surgical planning, while total body CT or PET‐CT can be used to rule out distant metastases.^12^ Accessible facial or cervical lesions may be analyzed with fine‐needle aspiration or open incisional biopsy. In many cases, the volume of tissue obtained from fine‐needle aspiration will not be suitable to reach histologic diagnosis, and a fine‐needle aspiration biopsy will not allow final diagnosis of the particular subtype of soft tissue sarcoma.

In the present case, an incisional biopsy was performed because the lesion had already ulcerated the skin. The procedure was carried out after MRI evaluation to obtain a tissue core and not only superficial tumor. Although in the literature incisional biopsy is reported to have an accuracy of 94% with no false positive diagnoses for UHPS,^13^ we prefer to perform a core biopsy in all lesions with subcutaneous growth to avoid disrupting the skin barrier which could facilitate dissemination of disease.

Until now, a standard therapeutic strategy has not been established, although surgical excision is usually considered the mainstay of treatment.^4^, ^6^, ^9^, ^13^ Wide resection with free margins is required, but a complete excision of head and neck localizations can be challenging due to the infiltrative nature and consequent invasion to critical structures. Regional metastases of head and neck sarcomas are almost exclusive of high‐grade lesions.^4^ In UHPS, lymph node metastases occur in 3%‐18% of cases and neck dissection is considered for advanced tumors or positive clinical lymph node metastases.^13^ In the presence of an advanced recurrence of high‐grade histology, the decision was to perform a selective neck dissection (levels Ib‐II‐III) in our patient rather than a modified radical neck dissection type III (functional) because it was a N0 tumor and the incidence of occult lymph node metastasis in this type of sarcoma is very rare.

Modern reconstruction surgery with microvascular free flaps enables wider excisions with good functionality and aesthetic results. Microvascular reconstruction in the elderly can be performed with a high rate of success. A recent review of the literature showed that there is no difference in terms of free flap success, surgical complications, and mortality rate between older and younger patients.^14^, ^15^

In this case, a latissimus dorsi free flap harvesting also the thoracodorsal nerve, which would have permitted to reconstruct the sacrificed facial nerve, could have been used for the reconstruction of the wide defect of parotid region. We preferred, however, to use as fasciocutaneus flap, a radial free flap in order to reconstruct the soft tissue defect without considering the reconstruction of facial nerve. The main reason for this choice was the possibility of working with a double team (demolitive/reconstructive) without the need to change the patient's surgical position therefore reducing duration of surgery. Furthermore, we choose not to proceed with the simultaneous reconstruction of the facial nerve considering the age of the patient and the severe prognosis of the lesion.

As an alternative to a simultaneous facial nerve reconstruction, a static suspension of the soft tissue of the middle third of the face and a static technique to help the eyelid closure is recommended in literature. This procedure was initially considered in this case but it was not performed because the patient's hemodynamic conditions did not allow the anesthesiological time to be further prolonged.

Adjuvant radiation therapy is generally recommended if margins are positive or close, if the lesion is high‐grade, in large tumors (more than 5 cm of diameter), or in the presence of metastatic lymph nodes.^5^ Unfortunately, postoperative radiotherapy, which improves local control, does not guarantee better survival due to the frequent development of distant metastases.^5^

The use of chemotherapy and/or radiotherapy as the primary treatment modality has been less successful; chemoresistance is not uncommon, and at present, there is no specific trial showing significant improvement of survival after adjuvant systemic chemotherapy.^5^, ^9^ The drugs most frequently used are the combination of doxorubicin and ifosfamide, but the benefit can be outweighed by associated toxicities, especially in older patients.^15^

Overall survival rates for UHPS range from 30% to 74%^(9)^. Positive or close surgical margins, after excision, correlate with an increased local recurrence rate and reduce overall survival.^4^ Male gender, advanced age, tumor size (>5 cm), lesion arising from bone, and deeper invasion are correlated with poor prognosis.^4^, ^5^ Most recurrences develop within 2 years after initial treatment.^5^, ^6^, ^8^

## 4 CONCLUSIONS

Undifferentiated high‐grade pleomorphic sarcoma is a slow‐growing tumor rarely localized in the head and neck region with potential nodal and distant metastases at the time of diagnosis. No specific guidelines for the management of this tumor have been established, but surgical excision with free margins associated with neck dissection in advanced cases in a multimodality regimen that currently represents the best chance of disease control. Postoperative radiotherapy improves local control, although neo‐adjuvant chemotherapy has been less investigated because it is limited by systemic toxicity. Free flap reconstruction makes more extensive resection possible and may improve local tumor control, while providing acceptable cosmetic and functional results.

## CONFLICT OF INTEREST

None declared.

## AUTHOR CONTRIBUTION

DT, MS, LORdZ, AZ: wrote this article.

DC, MS and DT: made operation.
